# Integrative analysis by targeted metabolomics revealed the regulatory function of donkey oil on skin metabolites

**DOI:** 10.3389/fmed.2025.1684399

**Published:** 2025-09-22

**Authors:** Min Li, Jinni Sun, Guangyuan Liu, Zhijie Chen, Tao Wu, Hang Tie, Cong Wang

**Affiliations:** ^1^Dong-E E-Jiao Co. Ltd., Liaocheng, China; ^2^Tianjin Key Laboratory of Food Biotechnology, School of Biotechnology and Food Science, Tianjin University of Commerce, Tianjin, China; ^3^Chinese Academy of Inspection and Quarantine Greater Bay Area, Zhongshan, China

**Keywords:** donkey oil, targeted metabolomics, skin metabolites, skin homeostasis, regulatory function

## Abstract

**Background:**

Donkey oil, an edible oil rich in unsaturated fatty acids and vitamin E, has the potential to become a multifunctional ingredient for skincare. However, its molecular mechanisms in maintaining skin health remain unknown.

**Methods:**

In this study, 64 volunteers with either healthy or inflammatory skin were divided into two groups and applied donkey oil for 28 days. Then, we measured the targeted metabolites including 65 kinds of organic acids, 94 kinds of amino acids, and 48 kinds of free fatty acids and their derivates in the skin by comparative metabolomics analysis of two groups to assess changes before and after application.

**Results:**

We found differential levels of seven (three upregulated and four downregulated) organic acids in the healthy skin group, six (two upregulated and four downregulated) organic acids in the inflammatory skin group, and one shared organic acid (4-hydroxyphenylacetic acid) in response to donkey oil application after 28 days. Regarding amino acids and their derivatives and free fatty acids, 10 (1 upregulated and 9 downregulated) amino acids and derivatives were found in the healthy skin group, while 7 (2 upregulated and 5 downregulated) were found in the inflammatory skin group. Additionally, three shared amino acids and their derivatives (5-hydroxytryptamine, tryptophan, and 5-aminovaleric acid) were found. For free fatty acids, 10 (8 upregulated and 2 downregulated) were regulated in the healthy skin group, and 7 (1 upregulated and 6 downregulated) were regulated in the inflammatory skin group. Furthermore, six shared free fatty acids were regulated by donkey oil.

**Conclusion:**

By metabolite functional annotation, donkey oil may influence the levels of several metabolites, including 4-hydroxyphenylacetic acid, 5-hydroxytryptamine, tryptophan, 5-aminovaleric acid, decanoic acid, octanoic acid, *cis*-11,14,17-eicosatrienoic acid, myristic acid, tridecanoic acid, and pentadecanoic acid. These metabolites are mainly enriched in aromatic amino acid metabolism and fatty acid biosynthesis metabolism to facilitate the maintenance of skin homeostasis.

## 1 Introduction

Donkey oil is an edible oil that holds importance in the fields of medicine and healthcare (1). It has been incorporated into ketogenic diets, which may help prevent tumor progression (2). Compared to conventional animal fats such as lard, beef tallow, and mutton tallow, donkey oil contains significantly higher levels of unsaturated fatty acids (UFAs) and essential fatty acids, including oleic acid (32.30%), linoleic acid (12.90%), and palmitic acid (26.33%) (3). In contrast, the fatty acid composition of fats from sheep, cattle, and horses is predominantly saturated. Importantly, bioactive fatty acids have been reported to play a critical role in inflammation (4). Notably, n-3 and n-6 polyunsaturated fatty acids (PUFAs) are closely related to the proliferation and differentiation of skin cells during the skin healing process (5). They could promote the processes of neovascularization, extracellular remodeling, migration, and cellular differentiation (6). Given this context, the UFA-rich profile of donkey oil exhibits its potential as an effective agent in skincare. In addition to fatty acids, donkey oil contains significantly higher levels of vitamin E (8.59 mg/100 g fat) compared to beef tallow and sheep fat (1). Vitamin E plays a crucial role in reducing lipid peroxidation and preventing skin photoaging (7). Collectively, these characteristics of donkey oil suggest its potential multifunctional benefits in skincare.

As the largest organ of the human body, the skin plays an essential role in defending against external damage, resisting microbial invasion, maintaining homeostasis, and preventing the loss of body fluids, electrolytes, and nutrients (8–10). Metabolic activities on the skin’s surface generate various metabolites, originating from sweat, sebum, and the degradation of outer skin proteins. The metabolites may also result from interactions between the host and the microorganisms that colonize the skin. Skin metabolites mainly include amino acids and their derivatives, peptides and proteins, carbohydrates, lipids, vitamins, pheromones, metal and non-metal ions, and xenobiotics (11). These metabolites are essential for maintaining skin health and homeostasis. Natural moisturizing factors (NMFs), such as L-serine, glutamic acid, L-alanine, lactic acid, and pyrrolidone-5-carboxylic acid, primarily consist of organic acids, amino acids, and fatty acids (12, 13). Furthermore, fatty acids possess antioxidant and antimicrobial properties, with metabolites like short-chain fatty acids (SCFAs) playing a significant role in modulating the skin barrier and immune function. For instance, propionic acid produced by *Cutibacterium acnes* promotes lipid synthesis and enhances UVB-induced melanin deposition (14).

In response to the growing trend toward minimalism and precision in the skincare industry, numerous horse oil and sheep oil skincare products have become available on the market. Donkey oil, rich in bioactive substances, shows potential as a multifunctional ingredient for maintaining healthy skin. In this study, we recruited both healthy-skinned volunteers and individuals with inflammatory skin conditions to apply donkey oil for 28 days. Through targeted comparative metabolomics, changes in the levels and pathway enrichment of three key skin surface metabolites—organic acids, amino acids, and fatty acids—were analyzed after applying donkey oil. By comparing the metabolic profiles of both groups after the application of donkey oil, we aimed to illustrate the potential mechanisms through which donkey oil keeps the skin in a stable condition and to provide a scientific basis for its use in the field of skincare.

## 2 Materials and methods

### 2.1 Chemicals

High-performance liquid chromatography (HPLC)-grade acetonitrile (ACN), methanol (MeOH), methyl tert-butyl ether (MTBE), and n-hexane were purchased from Merck (Darmstadt, Germany). MilliQ water (Millipore, Bradford, MA, USA) was used in all experiments. Ammonium acetate, formic acid, sodium chloride, and phosphate were bought from Sigma–Aldrich (St. Louis, MO, USA). All of the standards were also purchased from Sigma–Aldrich (St. Louis, MO, USA). The stock solutions of the standards were prepared at a concentration of 1 mg/mL in MeOH or MTBE, depending on the specific target metabolites. All stock solutions were stored at −20 °C. Before analysis, the stock solutions were diluted with MeOH or MTBE to create the working solutions.

### 2.2 Grouping of participants and collection of skin samples

Healthy volunteers (without preexisting dermatological conditions) and volunteers with inflammatory skin conditions (including acne, dermatitis, or eczema), aged 18–30 years with balanced gender distribution, were enrolled in the study. Volunteer groups are shown in Supplementary Table S1. Participants applied donkey oil to their facial regions (forehead and cheeks) twice daily (morning and evening) for 4 weeks, since the renewal cycle of skin cells is approximately 28 days (15, 16). Healthy samples were labeled as D0_H (pre-application) and D28_H (post-application). Inflammatory skin samples were labeled as D0_U (pre-application) and D28_U (post-application). Sampling was performed before application initiation [day 0 (D0)] and after 28 days of application [day 28 (D28)].

The skin samples were obtained using the classical swabbing method described as follows (17): the skin swab samples were collected using sterile nylon swabs pre-moistened with sterile saline. The swab was rubbed vertically across a 4 cm × 4 cm area on the forehead and cheeks 10 times with consistent pressure. The samples were collected before and after the application of donkey oil. The swab tips were placed into labeled sterile tubes and stored at −80 °C until analysis.

In this study, informed consent was obtained from all participating volunteers, and the study was conducted in accordance with the Declaration of Helsinki and was approved by the Institutional Review Board of the Chinese Academy of Inspection and Quarantine Greater Bay Area.

### 2.3 Pretreatment of skin samples and targeted metabolomics analysis based on UPLC-MS/MS or GC–MS/MS

The targeted metabolomics analysis consisted of several procedures, including the pretreatment and extraction of skin samples, metabolite detection, data preprocessing, quality control analysis, and target quantification. The extraction, identification, and quantification of the metabolites from the skin samples were carried out by Allwegene Technology Co., Ltd. (Beijing, China). The detailed procedure is provided in Supplementary files.

### 2.4 Statistical analysis

Unsupervised principal component analysis (PCA) was performed using the statistics function within R.^1^ The data were scaled to unit variance before unsupervised PCA. The results of the hierarchical cluster analysis (HCA) for samples and metabolites were presented as heatmaps with dendrograms. Significantly regulated metabolites between groups were determined by absolute Log_2_ fold change (Log_2_FC). Metabolites with a fold change of ≥2 or ≤0.5 between pre- and post-application samples were considered significantly altered. The identified metabolites were annotated using the Kyoto Encyclopedia of Genes and Genomes (KEGG) compound database.^2^ These annotated metabolites were then mapped to the KEGG pathway database.^3^ Pathways with significantly regulated metabolites were then mapped to and fed into metabolite set enrichment analysis (MSEA), and their significance was determined by *p*-values of hypergeometric tests (*p* ≤ 0.05).

## 3 Results

### 3.1 Overall metabolomics analysis of skin samples

Here, we applied targeted metabolomics to identify the changes in organic acids, amino acids and their derivatives, and free fatty acids in skin metabolites in response to donkey oil application. Overlay display analysis was performed using total ion flow plots of quality control (QC) samples to test the reproducibility of secondary metabolite extraction and detection. As shown in Supplementary Figures S1–S3, the total ion chromatogram (TIC) plots of the QC samples, the overlay analysis of samples, including organic acids (Supplementary Figure S1), amino acids and their derivatives (Supplementary Figure S2), and free fatty acids (Supplementary Figure S3), and multi-peak detection plots in positive and negative ion modes demonstrated a high level of reliability and reproducibility of the skin metabolites in response to donkey oil application.

### 3.2 Comparative metabolomics analysis of organic acids in two skin types after the application of donkey oil

**Figure 1:**
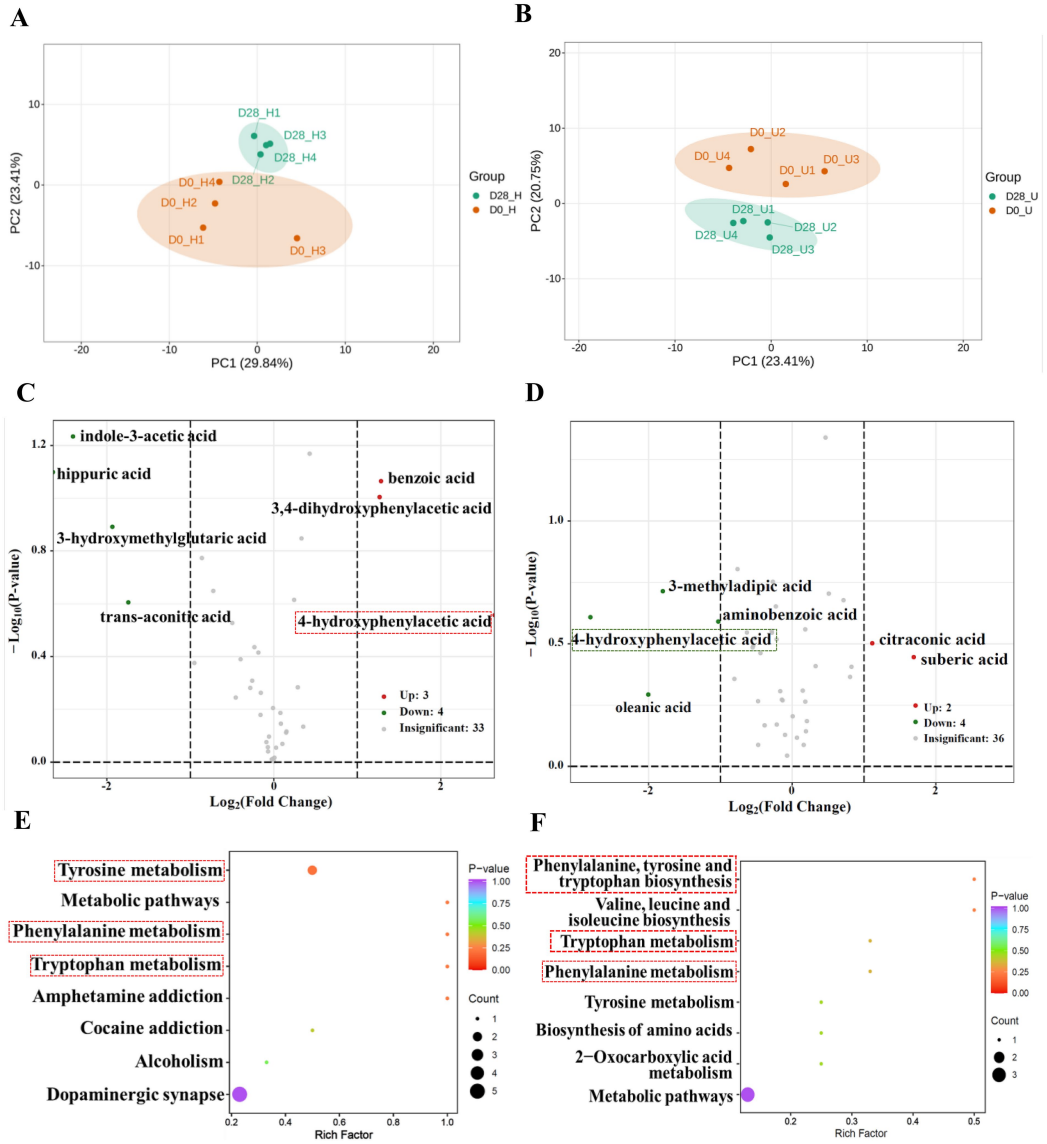
Metabolomic profiling of organic acids in skin samples before and after donkey oil application. Principal component analysis (PCA) of organic acids in healthy **(A)** and inflamed **(B)** skin before and after donkey oil treatment. Volcano plots showing significantly altered metabolites in healthy **(C)** and inflamed **(D)** skin groups following donkey oil treatment (red and green dots indicate upregulated and downregulated metabolites, respectively; gray dots represent the metabolites showing no significant difference). KEGG pathway enrichment analysis of differentially expressed organic acids in healthy **(E)** and inflamed **(F)** skin.

Targeted metabolomics possesses the advantages of performing qualitative and quantitative analyses of selected metabolites in biological samples through highly sensitive and specific analytical methods. To precisely explore the potential role of donkey oil in modulating skin organic acid metabolism, a total of 65 organic acids listed in Supplementary Table S2 were detected through targeted metabolomics analysis. By screening for the organic acids with a fold change of ≥2 or ≤0.5 between the donkey oil untreated and treated samples (18), seven organic acids in the healthy skin group, six organic acids in the inflammatory skin group, and one shared organic acid were identified as differential in response to donkey oil application, as listed in Supplementary Table S3. The PCA of the healthy skin group showed that principal component 1 (PC1) and principal component 2 (PC2) explained 29.84 and 23.41% of the total variance, respectively (Figure 1A). The PCA of the inflammatory skin group showed that PC1 and PC2 explained 23.41 and 20.75% of the total variance, respectively (Figure 1B). In addition, the scatter plot of these two groups clearly distinguished the before and after donkey oil-treated samples (Figures 1A,B). Subsequently, as shown in Figure 1C, the volcano plots demonstrated that the differential seven organic acids in the healthy group in response to donkey oil application included three upregulated organic acids, namely as benzoic acid, 4-hydroxybenzoic acid, and 3,4-dihydroxyphenylacetic acid (red dot), and four downregulated organic acids, namely trans-aconitic acid, indole-3-acetic acid, hippuric acid, and 3-hydroxymethylglutaric acid (green dot). In the inflammatory skin group after donkey oil application (Figure 1D), the differential six organic acids included two upregulated organic acids, namely suberic acid and citraconic acid (red dot), and four downregulated organic acids, namely oleanolic acid, 3-methyladipic acid, 4-hydroxyphenylacetic acid, and anthranilic acid (green dot). Although various organic acids were identified in response to donkey oil application between the healthy and inflamed skin groups, the KEGG enrichment analysis indicated a concentration of metabolic pathways, such as tryptophan metabolism, phenylalanine metabolism, and tyrosine metabolism (Figures 1E,F). This suggests that donkey oil might modulate skin metabolism, particularly in the metabolism of aromatic amino acids, thereby promoting skin health.

In terms of the functional annotation of the screened organic acids, our results indicated that benzoic acid was found to be upregulated, while its downstream metabolite, hippuric acid, was found to be simultaneously downregulated in the healthy skin group after donkey oil application. Benzoic acid is known as an aromatic carboxylic acid due to its antimicrobial properties and has been widely used in the therapy of acne (19); however, hippuric acid has been reported to accumulate at higher levels in individuals with various skin disorders compared to healthy controls, and excessive hippuric acid may contribute to oxidative stress (20). Therefore, we deduced that donkey oil might promote skin homeostasis by enhancing benzoic acid abundance and decreasing hippuric acid accumulation in the face. Indole-3-acetic acid is a known tryptophan-derived compound as an agonist of aryl hydrocarbon receptor (AhR), which is a regulator of skin barrier homeostasis (21). Previous research reported that indole-3-aldehyde, when applied to a mouse model of atopic dermatitis (AD)-like dermatitis, was effective in reducing inflammation via AhR (22). Therefore, the decrease in indole-3-acetic acid via tryptophan-dependent mechanisms after the application of donkey oil, as shown in the healthy group, might prevent skin inflammation.

In the inflammatory group, citraconic acid, a byproduct of citric acid metabolism, was significantly elevated after donkey oil application. Citraconic acid has been reported to exert anti-inflammatory effects (23). This upregulation of citraconic acid by donkey oil likely reflected the enhanced metabolic flux within the tricarboxylic acid (TCA) cycle, boosting cellular energy production to meet the increased metabolic demands of skin repair. Similarly, suberic acid, a medium-chain dicarboxylic acid (24), was upregulated after donkey oil application. As a metabolite linked to fatty acid oxidation and detoxification processes, upregulated levels of suberic acid by donkey oil might indicate its activated defense against anti-inflammation.

Interestingly, 4-hydroxyphenylacetic acid was the only shared organic acid in response to donkey oil in both the healthy and inflamed skin groups. However, 4-hydroxyphenylacetic acid was upregulated in the healthy skin group but downregulated in the inflammatory skin group after donkey oil application (Figures 1C,D and Supplementary Table S3). The log_2_FC value of 4-hydroxyphenylacetic acid in the healthy skin group after donkey oil application was infinite, indicating that its level was undetectable before the application. The log_2_FC value was −2.81 in the inflammatory skin group after donkey oil application (Supplementary Table S3, Supplementary Figure S4). These markedly opposing trends suggested that 4-hydroxyphenylacetic acid exhibited dose-dependent responses under different skin conditions, highlighting that skin health was regulated by systemic alterations in the skin metabolic network and confirming the advantage and potential of target metabolomics in depicting the complex of metabolites in skin homeostasis maintenance.

### 3.3 Comparative metabolomics analysis of amino acids in two skin types after the application of donkey oil

**Figure 2:**
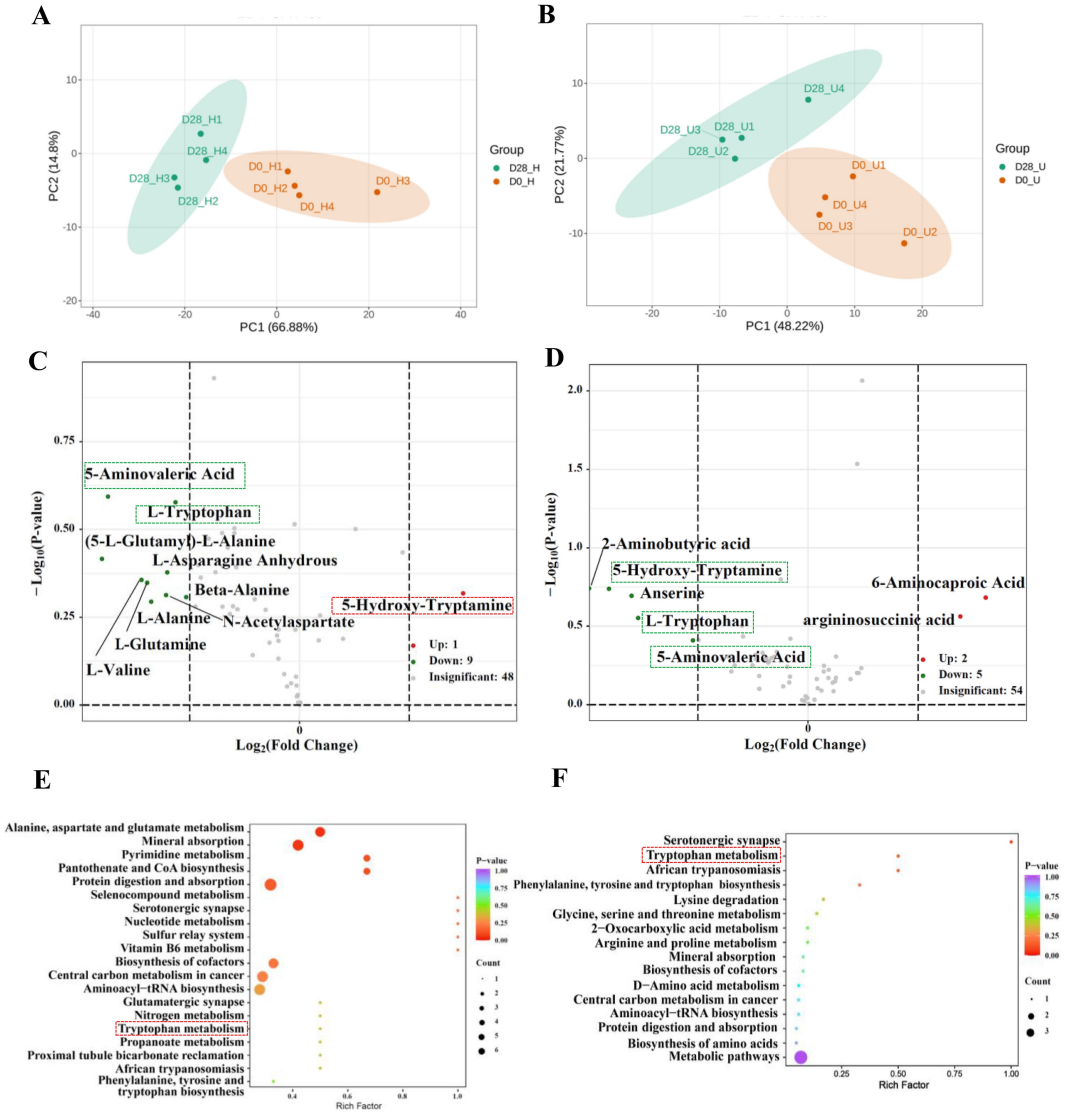
Integrated analysis of differential amino acids in healthy and inflamed skin before and after 28 days of donkey oil application. Principal component analysis (PCA) of amino acid profiles in the healthy group **(A)** and inflammation group **(B)**; Volcano plots showing significantly altered amino acids in the healthy group **(C)** and inflammation group **(D)** (red and green dots indicate upregulated and downregulated metabolites; gray dots represent the metabolites showing no significant difference); KEGG pathway enrichment analysis of differential amino acids in the healthy group **(E)** and inflammation group **(F)**.

A total of 94 amino acids were detected through targeted analysis (Supplementary Table S4). We screened for amino acids with a fold change of ≥2 or ≤0.5 between the untreated and treated donkey oil samples. This analysis identified 10 differential amino acids in the healthy skin group and 7 differential amino acids in the inflammatory skin group, as well as 3 shared amino acids and their derivatives in response to donkey oil application, as listed in Supplementary Table S5. The PCA of the healthy skin group showed that PC1 and PC2 explained 66.88 and 14.80% of the total variance, respectively (Figure 2A). The PCA of the inflammatory skin group showed that PC1 and PC2 explained 48.22 and 21.77% of the total variance, respectively (Figure 2B). In addition, the scatter plot of these two groups clearly distinguished the before and after donkey oil-treated samples (Figures 2A,B). The volcano plots in Figure 2C demonstrated that the differential 10 amino acids and their derivatives in the healthy group in response to donkey oil application included 1 upregulated amino acid and its derivatives, such as 5-hydroxytryptamine (red dot), and 9 downregulated amino acids and their derivates, namely N-acetylaspartate, (5-L-glutamyl)-L-alanine, L-valine, beta-alanine, L-asparagine anhydrous, L-alanine, L-glutamine, L-tryptophan, and 5-aminovaleric acid (green dot). However, in the inflammatory skin group after donkey oil application (Figure 2D), the differential seven amino acids and their derivatives included two upregulated amino acid and its derivatives, such as argininosuccinic acid, 6-aminocaproic acid (red dot), and five downregulated amino acids and its derivatives, namely L-tryptophan, 5-hydroxytryptamine, 5-aminovaleric acid, 2-aminobutyric acid, and anserine (green dot).

5-Hydroxytryptamine, tryptophan, and 5-aminovaleric acid were identified as the three shared amino acids and their derivatives in both healthy and inflamed skin groups after donkey oil application. Among these, tryptophan and 5-aminovaleric acid were all downregulated by donkey oil application in the healthy and inflamed skin groups. However, similar to the tendency of 4-hydroxyphenylacetic acid, 5-hydroxytryptamine was upregulated in the healthy group but downregulated in the inflammatory skin group after donkey oil application (Figures 2C,D, Supplementary Table S5, Supplementary Figure S5). 5-Hydroxytryptamine is both a neurotransmitter and an immune modulator and an important mediator of bidirectional interactions between the vasoactive amines and the skin (25). 5-Hydroxytryptamine induces fibrosis, although the mechanistic basis and growth factors regulating fibrosis and proliferation in the microenvironment are unclear (26). The upregulation of 5-hydroxytryptamine in the healthy skin group suggests that donkey oil could enhance neurotransmission, thereby helping to maintain healthy skin function and promote 5-hydroxytryptamine production. However, in the inflamed skin group, the significant downregulation of 5-hydroxytryptamine might effectively inhibit the fibrosis of the skin surface where there exists noticeable acne, dermatitis, and eczema, avoiding the inflamed spots from getting rough.

L-Tryptophan (Trp) is an essential amino acid, involved in several major metabolic pathways, including protein synthesis and conversion into kynurenine (Kyn). Kyn is subsequently transformed into various biologically active metabolites, including tryptamine, 5-hydroxytryptamine, kynurenic acid, picolinic acid, and NAD+, through quinolinic acid or anthranilic acid intermediaries, which play important roles in skin homeostasis maintenance (22, 27). Therefore, donkey oil prompted the conversion of Trp to supply more nutrients for more active skin cell regeneration, whether in the healthy skin or the inflamed skin group. Compared to these two well-depicted substances, the role of 5-aminovaleric acid in skincare remains unclear and requires further exploration.

Although the KEGG pathways related to amino acid metabolites after donkey oil application were more diversified and dispersed between the healthy and inflamed skin in comparison with those KEGG pathways of organic acids, the KEGG analysis also showed the enriched common pathways of aromatic amino acid metabolism (Figures 2E,F).

### 3.4 Comparative metabolomics analysis of free fatty acids in two skin types after application of donkey oil

**Figure 3:**
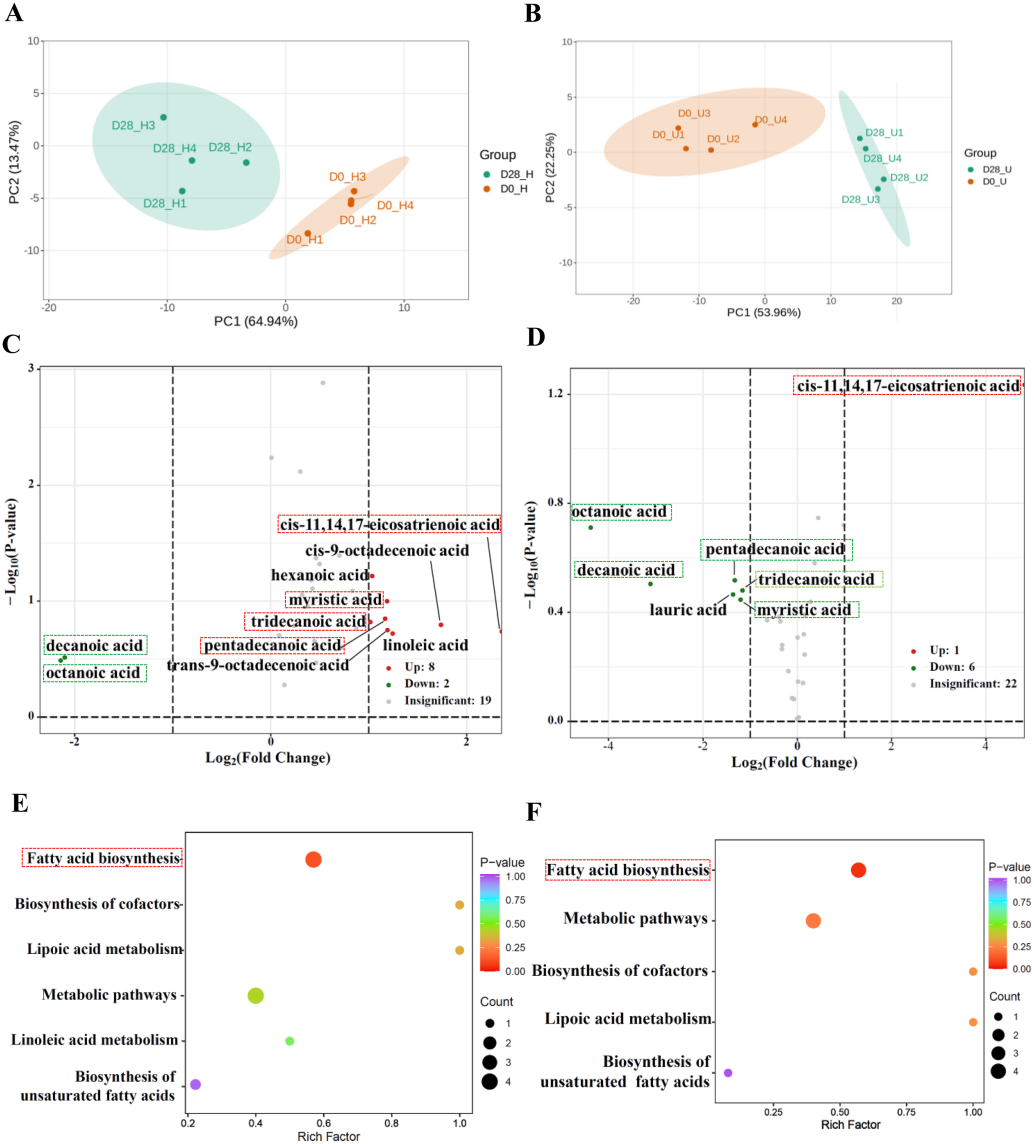
Integrated analysis of differential free fatty acids in healthy and inflamed skin before and after 28 days of donkey oil application. Principal component analysis (PCA) of fatty acid profiles in the healthy group **(A)** and inflammation group **(B)**; Volcano plots showing significantly altered fatty acids in the healthy group **(C)** and inflammation group **(D)** (red, and green dots indicate upregulated, downregulated metabolites; gray dots represent the metabolites showing no significant difference); KEGG pathway enrichment analysis of differential free fatty acids in the healthy group **(E)** and inflammation group **(F)**.

A total of 48 free fatty acids were detected through targeted analysis in this section (Supplementary Table S6). By screening for free fatty acids with a fold change of ≥2 or ≤0.5 between the donkey oil-treated and untreated samples, we identified differential 10 and 7 amino acids in the healthy and inflammatory skin groups, respectively, and 6 shared free fatty acids and their derivatives in response to donkey oil application, as listed in Supplementary Table S7. The PCA of the healthy skin group showed that PC1 and PC2 explained 64.94 and 13.47% of the total variance, respectively (Figure 3A). The PCA of the inflammatory skin group showed that PC1 and PC2 explained 53.96 and 22.25% of the total variance, respectively (Figure 3B). In addition, the scatter plot of these two groups clearly distinguished the before and after of donkey oil application (Figures 3A,B). The volcano plots in Figure 3C demonstrated that the differential 10 free fatty acids in the healthy skin group in response to donkey oil application included 8 upregulated fatty acids, namely *cis*-11,14,17-eicosatrienoic acid, tridecanoic acid, myristic acid, pentadecanoic acid, hexanoic acid, *cis*-9-octadecenoic acid, linoleic acid and trans-9-octadecenoic acid (red dot), and two downregulated fatty acids, namely decanoic acid and octanoic acid (green dot). However, in the inflammatory skin group after donkey oil application (Figure 3D), the differential seven fatty acids included one upregulated fatty acid, namely *cis*-11,14,17-eicosatrienoic acid (red dot), and six downregulated amino acids, namely decanoic acid, octanoic acid, lauric acid, myristic acid, tridecanoic acid, and pentadecanoic acid (green dot). Among these, six shared fatty acids, namely decanoic acid, octanoic acid, *cis*-11,14,17-eicosatrienoic acid, myristic acid, tridecanoic acid, and pentadecanoic acid, were found in response to donkey oil application, whether in the healthy or the inflamed skin group.

To the aspect of the six shared fatty acids functional annotation, our results showed decanoic acid and octanoic acid were downregulated, while *cis*-11,14,17-eicosatrienoic acid was upregulated by donkey oil in both healthy and inflamed groups. Myristic acid, tridecanoic acid, and pentadecanoic acid were upregulated in the healthy group but downregulated in the inflamed group (Figures 3C,D, Supplementary Table S7). Since decanoic acid and octanoic acid may not be inherent metabolites of sebaceous gland cells and are likely derived from microbial metabolism on the skin surface (28), the decreased trends of them suggested that donkey oil might affect the skin surface microbiota and suppress the secretion of decanoic and octanoic acids. *Cis*-11,14,17-eicosatrienoic acid has been reported to play key roles in antiaging and protecting the skin barrier (29, 30). After applying donkey oil, its levels in both skin types were enhanced, which might be due to its role in protecting the skin barrier. In addition, although myristic acid and pentadecanoic acid have been reported to show anti-inflammatory features (31) and contribute to maintaining microbial homeostasis (32), the log_2_FC values of myristic acid and pentadecanoic acid were 1.19 and 1.17 in the healthy group and −1.20 and −1.33 in the inflammatory group, respectively (Supplementary Table S7, Supplementary Figure S6). This significantly opposite trend suggested that myristic acid and pentadecanoic acid exhibited dose-dependent responses under various skin conditions. Compared to these two substances, the role of tridecanoic acid in skincare remains unclear and requires further exploration.

Although distinct fatty acids were identified in the healthy and inflamed skin groups following donkey oil treatment, the KEGG enrichment analysis showed that the metabolic changes in both groups converged on pathways like Fatty acid biosynthesis, Biosynthesis of cofactors, and metabolic pathways (Figures 3E,F). This suggests that the donkey oil might modulate the skin metabolism through Fatty acid synthesis to enhance skin health.

## 4 Discussion

In this study, skin samples were collected from volunteers with healthy skin and those with inflammatory skin conditions, both before and after the application of donkey oil. Targeted metabolomics was employed to conduct a comprehensive analysis of the skin samples, allowing us to identify the differential organic acids, amino acids, and free fatty acids associated with various skin conditions in response to the application of donkey oil for 28 days. The metabolic pathways involved were predicted by KEGG analysis based on the dynamic changes in metabolites. This analysis provides valuable insights into how donkey oil may exert its protective effects on the skin through the regulation of organic acids, amino acids, and free fatty acids.

The present study has several strengths: the inclusion of individuals with healthy skin and inflamed skin; reliable qualitative and quantitative methods for the analysis of metabolites using targeted metabolomics; and comprehensive measurement of organic acids, amino acids and their derivatives, and fatty acids. Our findings demonstrated the significant value of donkey oil in skin homeostasis maintenance by modulating distinct metabolites under healthy and inflamed skin conditions. KEGG pathway analysis enriched the changes of metabolites from these three categories mainly to the aromatic amino acid metabolism pathway, which involves tryptophan, phenylalanine, and tyrosine metabolism, as well as fatty acid metabolism. One limitation of our study is that the sample size was 32 volunteers and the number of samples was relatively small, meaning the large diversity of skin conditions with respect to gender, age, geographical location, habit, etc., could not be represented and partially led to the low predictive accuracy for some potential metabolites. Therefore, we sub-grouped these 32 volunteers into 4 groups to maximize the repeatability, and the PCA analysis of these three kinds of metabolites all showed distinguished variance in each group before and after donkey oil treatment, confirming the reliability of this study (Figures 2–4). Moreover, our previous results have indicated that donkey oil possessed organic acids such as lactic acid, salicylic acid, L-malic acid 4-aminobutyric acid, pyroglutamic acid, sebacic acid, and taurine and fatty acids such as palmitic acid, *γ*-linolenic acid, linoleic acid, *cis*-9-octadecenoic acid, stearic acid, arachidonic acid, and amino acid and its derivates 2-aminoethanesulfonic acid, L-serine, L-tryptophan, L-phenylalanine, and L-tyrosine (33). However, the 10 metabolites responding to donkey oil in our present study, namely 4-hydroxyphenylacetic acid, 5-hydroxytryptamine, 5-aminovaleric acid, tryptophan, *cis*-11,14,17-eicosatrienoic acid, decanoic acid, octanoic acid, myristic acid, tridecanoic acid, and pentadecanoic acid, seemed to be unresponsive to the donkey oil during the sampling process because tryptophan, decanoic acid, and octanoic acid, which are present in donkey oil, were all downregulated, thus excluding interference from donkey oil in the targeted metabolomics analysis (Supplementary Tables S3, S5, and S7) and further confirming the reliability of this study.

Changes to the shared metabolites in different skin types may better reflect the regulatory mechanisms by which donkey oil modulates skin homeostasis. The consistent trends of change indicated a unidirectional regulatory effect by donkey oil. In contrast, opposite trends indicated that the functions of these particular metabolites on skin might be dose-dependent or influenced by the broader metabolic network. Specifically, the only shared organic acid, 4-hydroxyphenylacetic acid, exhibited a group-specific response to donkey oil application: it was significantly upregulated in the healthy skin group but downregulated in the inflamed skin group (Figures 1C,D). As a microbial metabolite of tyrosine (34), it has been reported to attenuate inflammatory damage and inhibit mitogen-activated protein kinase (MAPK) signaling, thereby suppressing inflammatory cytokine production (35, 36). Thus, its elevation in the healthy group might reflect enhanced microbial metabolic activity and redox homeostasis, whereas its reduction in the inflamed group indicated impaired microbial resilience under inflammatory stress. Among the shared amino acids, 5-hydroxytryptamine (one of the downstream metabolites of tryptophan) and tryptophan drew particular attention. Notably, the distinct trends of 5-hydroxytryptamine across different skin types not only highlighted its potential role in healthy skin but also reflected the relative complexity of metabolic regulation in inflamed skin. The downregulation of tryptophan in both skin types further supported previous reports suggesting that skin homeostasis requires relatively low levels of amino acids (37). Although 5-hydroxytryptamine was reported to exert its biological functions largely through regulation by the gut microbiota (38), our findings implied a close association between tryptophan metabolism and skin metabolism. Among the shared free fatty acids, similar to 4-hydroxyphenylacetic acid, myristic acid, tridecanoic acid, and pentadecanoic acid were significantly upregulated in the healthy skin group but downregulated in the inflamed skin group (Figures 3C,D), supporting the condition-dependent metabolic responses of skin.

The intrinsic properties and metabolic origins of different metabolites may suggest their potential enrichment in specific metabolic pathways. For instance, benzoic acid is derived from phenolic acid (39), 4-hydroxyphenylacetic acid is the metabolite of tyrosine (34), and 5-hydroxytryptamine and indole-3-acetic acid are both the downstream metabolites of tryptophan (40). These characteristics suggested a likely enrichment of pathways involved in aromatic amino acid metabolism, which was supported by KEGG enrichment analyses (Figures 1E, 2E), highlighting the interrelation between organic acids and amino acids. In addition, fatty acid biosynthesis pathways were significantly enriched regardless of skin conditions (Figures 3E,F). This finding further showed that donkey oil played a key role in modulating fatty acid metabolism on the skin surface.

In summary, our results suggest that donkey oil maintains skin homeostasis primarily by modulating both host skin cell and skin microbiota metabolism, especially through the regulation of aromatic amino acid metabolism and fatty acid biosynthesis and potentially neurochemical signaling. Our findings supported the potential of donkey oil as a multifunctional agent in skin health. In the future, the combined analysis of the skin microbiome and metabolome will reveal deeper mechanisms by which donkey oil modulates skin homeostasis.

## Data Availability

The original contributions presented in the study are included in the article/Supplementary material, further inquiries can be directed to the corresponding authors.

## Footnotes

^1^ www.r-project.org

^2^ http://www.kegg.jp/kegg/compound/

^3^ http://www.kegg.jp/kegg/pathway.html
