# Supplementary material for: Integrative analysis by targeted metabolomics revealed the regulatory function of donkey oil on skin metabolites
Source: Front Med (Lausanne). 2025 Sep 22;12:1684399. doi: 10.3389/fmed.2025.1684399 (PMC12497717; doi:10.3389/fmed.2025.1684399)
Supplement: Supplementary file 1 [file Table_1.docx]

Supplementary Material

**Integrative Analysis by Targeted Metabolomics Revealed the Regulatory Function of Donkey Oil on Skin Metabolites**

# Supplementary methods

**1. Sample Preparation and** **Extraction for Detecting Organic Acids and Amino Acids**

After the samples were thawed, the samples were cut into small pieces and mixed with 3000 μL of 70% methanol/water respectively. The samples were vortexed for 5 min under the condition of 2500 r/min and placed on ice for 15 min centrifuged at 12000 r/min for 3 min at 4℃. Took 1mL of supernatant into the corresponding 1.5 mL centrifuge tube and concentrated it until it was completely dry. Finally, reconstituted with 200 μL of 70% methanol/water, vortexed for 5 min and placed the supernatant in -20℃ refrigerator for 30 min. Then the supernatant was centrifuged again at 12000 r/min for 20 min at 4℃. After centrifugation, transferred 80 μL of supernatant for further LC-MS/MS analysis.

**1.1 LC Conditions** **for Detecting Organic Acids and Amino Acids**

Targeting for organic acids, 0.05% formic acid in water and 0.05% formic acid in acetonitrile were used as mobile phases A and B, respectively. The elution was carried out as follows: 5% B (0 min), increased to 95% B (8-9.5 min), finally ramped back to 5% B (9.6-12 min). The flow rate was set at 0.35 mL/min, the column temperature at 40 ℃ and the injection volume at 2 μL.

Targeting for amino acids, 0.04% formic acid in water with 2 mM ammonium acetate and 0.04% formic acid in acetonitrile with 2 mM ammonium acetate were used as mobile phases A and B, respectively. The elution was carried out as follows: 90% B (0-1.2 min), decreased to 60% B (9 min), 40% B (10-11 min), finally ramped back to 90% B (11.01-15 min); The flow rate was set at 0.35 mL/min, the column temperature at 40 ℃ and the injection volume at 2 μL.

**1.2 UPLC Conditions for Detecting Organic Acids and Amino Acids**

The samples were analyzed by ultra-performance liquid chromatography (UPLC) (Exi on LC™ AD, Foster City, CA, USA, https://sciex.com.cn/) and tandem mass spectrometry (MS/MS) (Applied Biosystems 6500 QTRAP, Foster City, CA, USA, https://sciex.com.cn/) using ACQUITY HSS T3 column (Waters, 2.1×100 mm, 1.8 μm). The LC analytical conditions targeting for organic acids and amino acids were detailed in supplementary methods respectively.

**1.3 ESI-MS/MS Conditions for Detecting Organic Acids and Amino Acids**

The effluent was alternatively connected to an ESI-triple quadrupole-linear ion trap (Q TRAP)-MS. LIT and triple quadrupole (QQQ) scans were acquired on a triple

quadrupole-linear ion trap mass spectrometer (Q TRAP), API 6500 QTRAP LC/MS/MS System, equipped with an ESI Turbo Ion-Spray interface, operating in a positive ion mode and controlled by Analyst 1.6.3 software (AB Sciex). The mass spectrometry data were collected in positive/negative electrospray ionization (ESI) mode with the ESI source at 550℃ and using an ionization voltage of 5500 V (positive ionization mode)/-4500 V (negative ionization mode) and controlled by Analyst 1.6 software (AB Sciex); curtain gas (CUG) was set at 35 psi; the declustering potential (DP) and collision energy (CE) for individual Multi Reaction Monitoring (MRM) transitions were optimized. A specific set of MRM transitions were monitored for each period according to the organic acid/ amino acid eluted within this period.

**2.Skin Samples Pretreatment and Targeted Metabolomics Analysis of Free Fatty Acids Based on GC-MS/MS**

After the samples were thawed, an amount of 0.05 g of each sample was weighed into the tube with 150 μL MeOH, 200 μL MTBE, 50 μL 36% phosphoric acid/water (v/v, precooled at -20℃) added. The samples were vortexed for 3 min followed ultrasonated for 10 min and centrifuged for 5 min at 4℃ and 12000 r/min. 200 μL of supernatant was concentrated on a nitrogen blower until dry. 300 μL of 15% boron trifluoride methanol solution was injected into the dried samples followed vortexing for 3 minutes and keeping in an incubator for 30 min at 60℃ to harvest the derivatization. Accurately adding 500 μL of n-hexane solution and 200 μL of saturated sodium chloride solution into the derivative solution after cooling to room temperature. The mixed liquid was vortexed for 3 min and centrifuged for 5 min at 4℃ and 12000 r/min. The upper n-hexane layer solution was transferred into GC injection vial for analysis.

**2.1 GC Conditions for Detecting Free Fatty Acids**

The GC analytical conditions were as follows using a gas chromatography (Agilent 8890, https://Agilent.com.cn/) couple to triple quadrupole mass spectrometry (Agilent 7000E, <https://Agilent.com.cn/>). The separation was conducted on a DB-5MS capillary column (30 m×0.25 mm×0.25 μm, Agilent) with high purity helium (purity >99.999%) as carrier gas. The heating procedure was started at 50℃ and held for 1.5 min, increased to 170℃ at a rate of 30℃/min, increased to 200℃ at a rate of 15℃/min, increased to 220℃ at a rate of 5℃/min and held for 2 min, increased to 228℃ at a rate of 8℃/min, increased to 240℃ at a rate of 20℃/min and held for 2 min, increased to 265℃ at a rate of 25℃/min and held for 2 min, increased to 280℃ at a rate of 30℃/min and held for 2 min. The flow rate was set at 1 mL/min. The injection volume was set at 1 μL with splitless. The inlet temperature was set at 250℃.

**2.2 The** **Mass Spectrometry Parameters**

The temperature and ionization voltage of electron ionization source (EI source) was 230 ℃ and 70 eV, respectively. The transmission line and quadrupole temperature were 280 ℃ and 150 ℃. The sample data was acquired in SIM mode after solvent delay for 4 min.

# Supplementary Figures and Tables

**Table S1.** Study design and participants grouping.

| Healthy skin volunteers (n=32) | | | | Inflammatory skin volunteers (n=32) | | | |
| --- | --- | --- | --- | --- | --- | --- | --- |
| Pre-application  (D0) | | Post-application (D28) | | Pre-application  (D0) | | Post-application (D28) | |
| Group | No. | Group | No. | Group | No. | Group | No. |
| H0_1 | H0_11 | H28_1 | H28_11 | U0_1 | U0_11 | U28_1 | U28_11 |
|  | H0_12 |  | H28_12 |  | U0_12 |  | U28_12 |
|  | H0_13 |  | H28_13 |  | U0_13 |  | U28_13 |
|  | H0_14 |  | H28_14 |  | U0_14 |  | U28_14 |
|  | H0_15 |  | H28_15 |  | U0_15 |  | U28_15 |
|  | H0_16 |  | H28_16 |  | U0_16 |  | U28_16 |
|  | H0_17 |  | H28_17 |  | U0_17 |  | U28_17 |
|  | H0_18 |  | H28_18 |  | U0_18 |  | U28_18 |
| H0_2 | H0_21 | H28_2 | H28_21 | U0_2 | U0_21 | U28_2 | U28_21 |
|  | H0_22 |  | H28_22 |  | U0_22 |  | U28_22 |
|  | H0_23 |  | H28_23 |  | U0_23 |  | U28_23 |
|  | H0_24 |  | H28_24 |  | U0_24 |  | U28_24 |
|  | H0_25 |  | H28_25 |  | U0_25 |  | U28_25 |
|  | H0_26 |  | H28_26 |  | U0_26 |  | U28_26 |
|  | H0_27 |  | H28_27 |  | U0_27 |  | U28_27 |
|  | H0_28 |  | H28_28 |  | U0_28 |  | U28_28 |
| H0_3 | H0_31 | H28_3 | H28_31 | U0_3 | U0_31 | U28_3 | U28_31 |
|  | H0_32 |  | H28_32 |  | U0_32 |  | U28_32 |
|  | H0_33 |  | H28_33 |  | U0_33 |  | U28_33 |
|  | H0_34 |  | H28_34 |  | U0_34 |  | U28_34 |
|  | H0_35 |  | H28_35 |  | U0_35 |  | U28_35 |
|  | H0_36 |  | H28_36 |  | U0_36 |  | U28_36 |
|  | H0_37 |  | H28_37 |  | U0_37 |  | U28_37 |
|  | H0_38 |  | H28_38 |  | U0_38 |  | U28_38 |
| H0_4 | H0_41 | H28_4 | H28_41 | U0_4 | U0_41 | U28_4 | U28_41 |
|  | H0_42 |  | H28_42 |  | U0_42 |  | U28_42 |
|  | H0_43 |  | H28_43 |  | U0_43 |  | U28_43 |
|  | H0_44 |  | H28_44 |  | U0_44 |  | U28_44 |
|  | H0_45 |  | H28_45 |  | U0_45 |  | U28_45 |
|  | H0_46 |  | H28_46 |  | U0_46 |  | U28_46 |
|  | H0_47 |  | H28_47 |  | U0_47 |  | U28_47 |
|  | H0_48 |  | H28_48 |  | U0_48 |  | U28_48 |


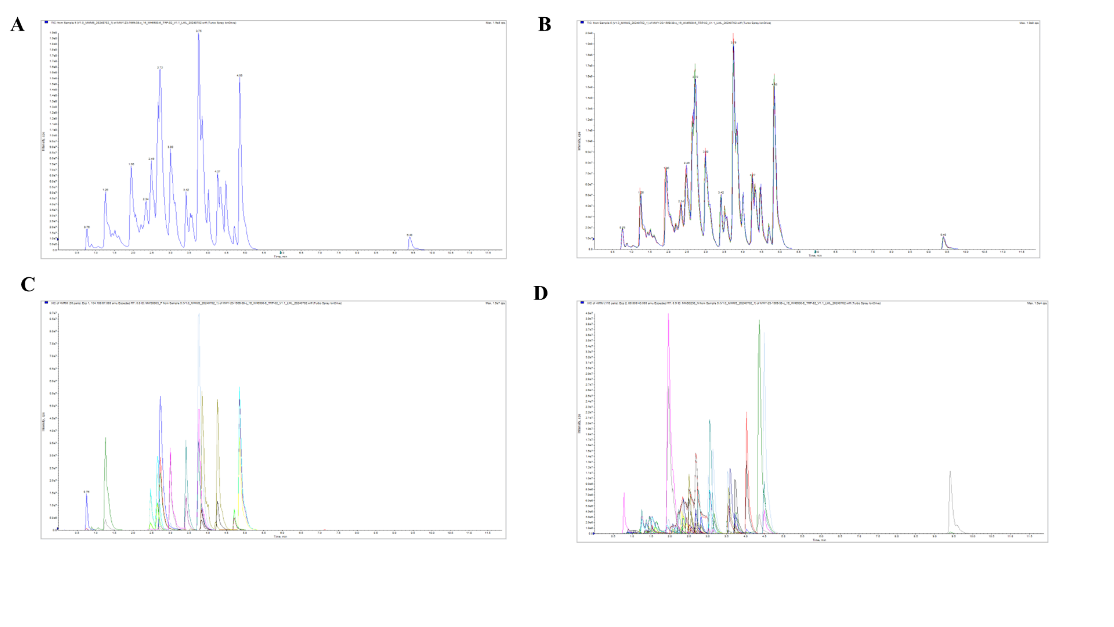


**Figure S1.** The overlay analysis of the QC-TIC diagram (A-B) and the sample multi-peak detection diagram under both positive and negative ion modes (C-D) targeting organic acids.

**
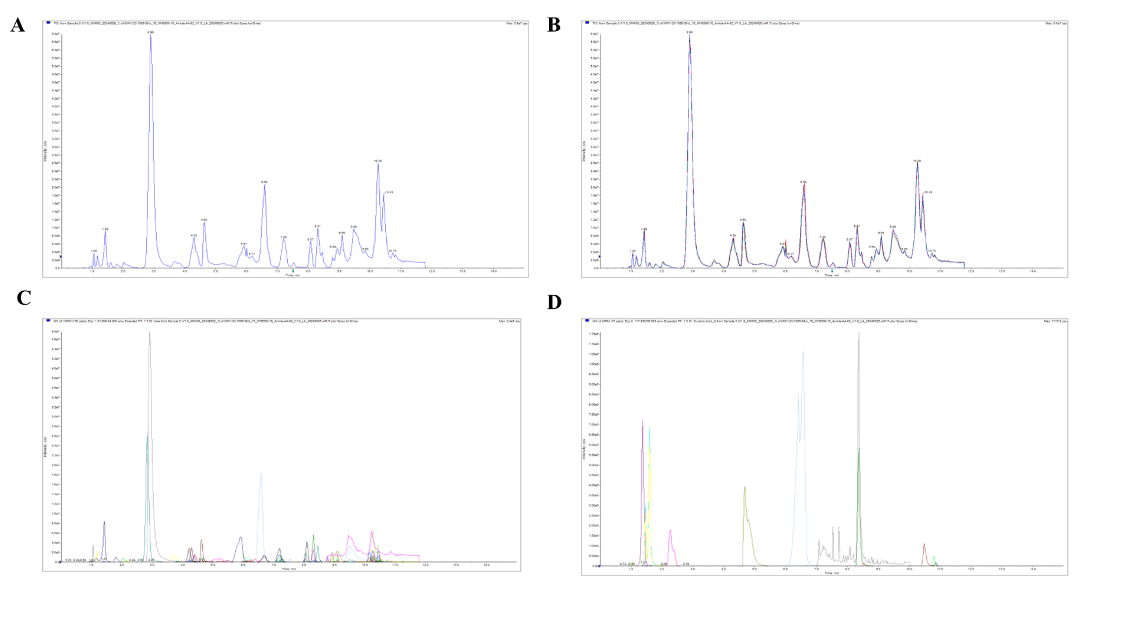
**

**Figure S2.** The overlay analysis of the QC-TIC diagram (A-B) and the sample multi-peak detection diagram under both positive and negative ion modes (C-D) targeting amino acids.

**
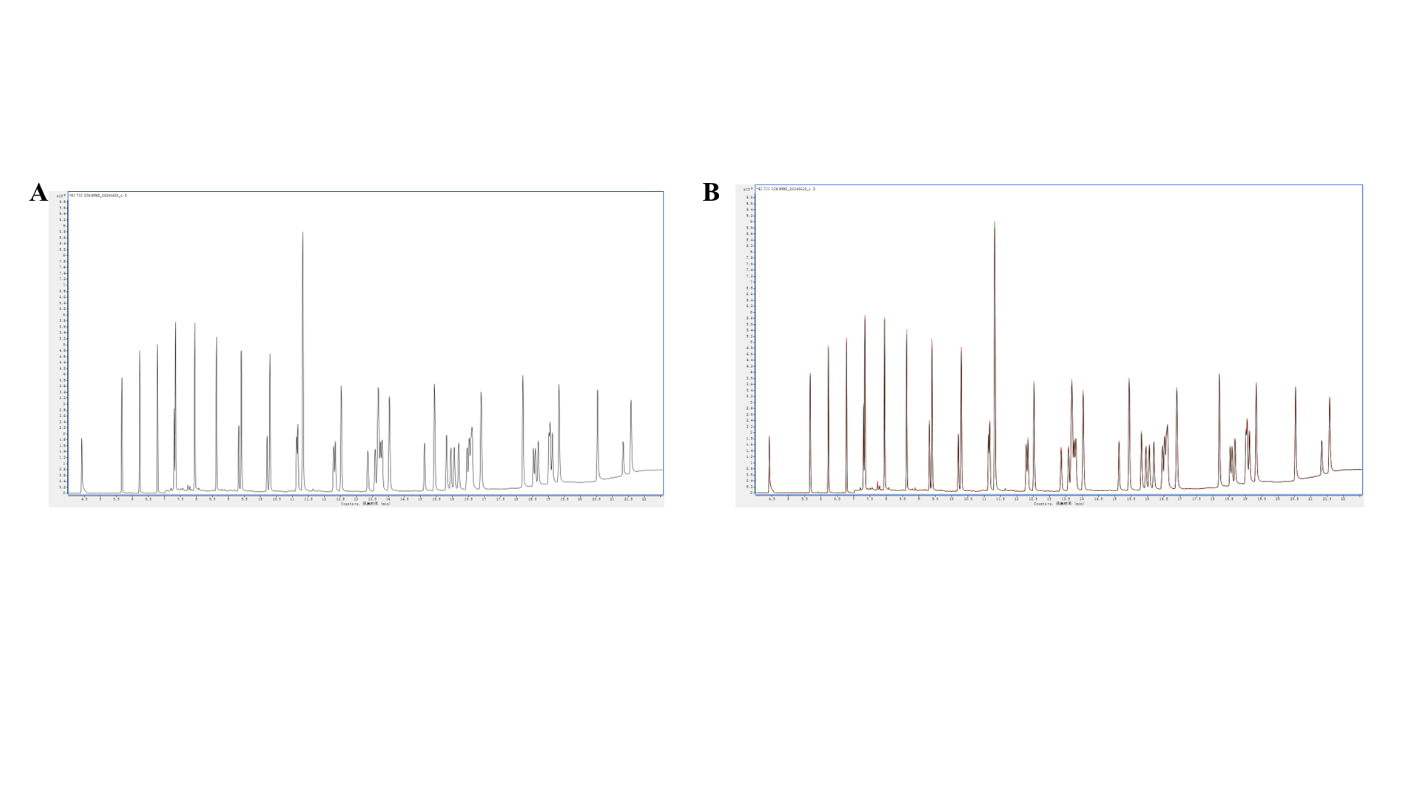
**

**Figure S3.** The overlay analysis of the QC-TIC diagram (A-B) targeting free fatty acids.

**Table S2.** **All detected organic acids in this study.**

| No. | Name | No. | Name |
| --- | --- | --- | --- |
| 1 | indolelactic acid | 34 | pyruvic acid |
| 2 | kynurenic acid | 35 | trans-aconitic acid |
| 3 | phenaceturic acid | 36 | homovanillic acid |
| 4 | cinnamic acid | 37 | hippuric acid |
| 5 | maslinic acid | 38 | shikimic acid |
| 6 | 2-indolecarboxylic acid | 39 | kynurenine |
| 7 | 5-hydroxyindoleacetic acid | 40 | 2-hydroxyphenylacetic acid |
| 8 | 4-aminobutyric acid | 41 | 4-hydroxyphenylacetic acid |
| 9 | aminobenzoic acid | 42 | lactic acid |
| 10 | indole-3-acetic acid | 43 | methylmalonic acid |
| 11 | 3-hydroxyphenylacetic acid | 44 | succinic acid |
| 12 | 3-hydroxyhippuric acid | 45 | 2-hydroxy-2-methylbutyric acid |
| 13 | 4-hydroxyhippuric acid | 46 | 2-hydroxyisovaleric acid |
| 14 | oleanic acid | 47 | 3-hydroxyisovaleric acid |
| 15 | pyroglutamic acid | 48 | 2-methylsuccinic acid |
| 16 | 3-methyladipic acid | 49 | glutaric acid |
| 17 | sebacic acid | 50 | ethylmalonic acid |
| 18 | benzenepropanoic acid | 51 | adipic acid |
| 19 | 3-hydroxymethylglutaric acid | 52 | oxoglutaric acid |
| 20 | 4-coumaric acid | 53 | neochlorogenic acid |
| 21 | pantothenic acid | 54 | cryptochlorogenic acid |
| 22 | caffeic acid | 55 | fumaric acid |
| 23 | maleic acid | 56 | levulinic acid |
| 24 | gallic acid | 57 | cis-aconitic acid |
| 25 | azelaic acid | 58 | citraconic acid |
| 26 | carnosic acid | 59 | salicylic acid |
| 27 | suberic acid | 60 | 4-hydroxybenzoic acid |
| 28 | taurine | 61 | 3,4-dihydroxyphenylacetic acid |
| 29 | 3-hydroxyphenyl-hydracrylic acid | 62 | hydroxyphenyllactic acid |
| 30 | 3-D-hydroxybutyric acid | 63 | 5-hydroxymethyl-2-furoic acid |
| 31 | 3-phenyllactic acid | 64 | L-malic acid |
| 32 | ferulic acid | 65 | tartaric acid |
| 33 | benzoic acid |  |  |

**Table S3.** Differential Changed Organic Acids in Healthy and Inflammatory Skin Samples.

| Name | Healthy Samples  (D28_H vs D0_H) | | | Inflammatory Samples  (D28_U vs D0_U) | | | |
| --- | --- | --- | --- | --- | --- | --- | --- |
|  | **FC** | **Log_2_FC** | **Type** | **FC** | | **Log_2_FC** | **Type** |
| benzoic acid | 2.43 | 1.28 | ↑ | |  |  |  |
| 3,4-dihydroxyphenylacetic acid | 2.41 | 1.26 | ↑ | |  |  |  |
| hippuric acid | 0 | -Inf | ↓ | |  |  |  |
| indole-3-acetic acid | 0.19 | -2.40 | ↓ | |  |  |  |
| trans-aconitic acid | 0.29 | -1.74 | ↓ | |  |  |  |
| 3-hydroxymethylglutaric acid | 0.26 | -1.93 | ↓ | |  |  |  |
| 4-hydroxyphenylacetic acid | Inf | Inf | ↑ | | 0.14 | -2.81 | ↓ |
| oleanic acid |  |  |  | | 0.25 | -2.00 | ↓ |
| aminobenzoic acid |  |  |  | | 0.28 | -1.80 | ↓ |
| 3-methyladipic acid |  |  |  | | 0.49 | -1.03 | ↓ |
| citraconic acid |  |  |  | | 2.17 | 1.12 | ↑ |
| suberic acid |  |  |  | | 3.23 | 1.69 | ↑ |

*****FC means Fold changes

**
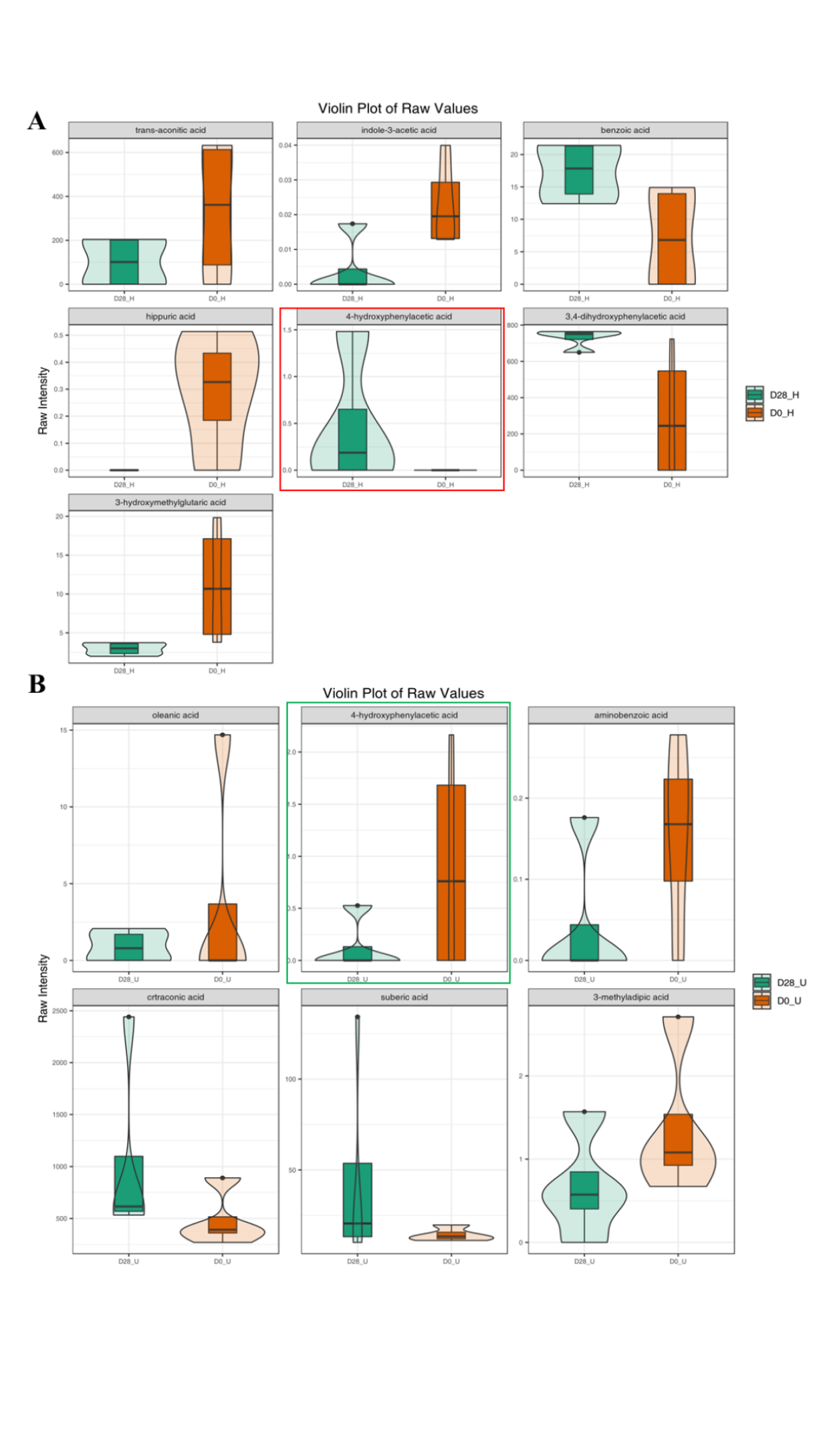
**

**Figure S4.** Volin plots of raw values of differential organic acids in healthy (A) and inflamed (B) skin before and after donkey oil treatment.

**Table S4.** All detected amino acids in this study.

| **No.** | | **Name** | **No.** | **Name** | **No.** | | **Name** |
| --- | --- | --- | --- | --- | --- | --- | --- |
| 1 | 2-Aminoethanesulfonic Acid | | 32 | Beta-Alanine | 63 | Nα-Acetyl-L-Arginine | |
| 2 | L-Cystine | | 33 | Sarcosine | 64 | 1-Methylhistidine | |
| 3 | 1,3-Dimethyluric Acid | | 34 | L-Pipecolic Acid | 65 | γ-Glutamate-Cysteine | |
| 4 | N-Propionylglycine | | 35 | L-Theanine | 66 | Nα-Acetyl-L-glutamine | |
| 5 | N-Isovaleroylglycine | | 36 | Ethanolamine | 67 | N-Acetyl-L-Tyrosine | |
| 6 | Succinic Acid | | 37 | 3-N-Methyl-L-Histidine | 68 | γ-Aminobutyric Acid | |
| 7 | 5-Hydroxy-tryptophan | | 38 | Homoserine | 69 | D-Alanyl-D-Alanine | |
| 8 | 3,7-Dimethyluric Acid | | 39 | Creatine | 70 | Guanidinoethyl Sulfonate | |
| 9 | Glycine | | 40 | kynurenine | 71 | Homo-L-arginine | |
| 10 | L-Alanine | | 41 | L-Cystathionine | 72 | L-Tryptophyl-L-glutamic acid | |
| 11 | L-Valine | | 42 | 5-Aminovaleric Acid | 73 | Nicotinuric Acid | |
| 12 | L-Leucine | | 43 | N6-Acetyl-L-Lysine | 74 | N-Acetylneuraminic Acid | |
| 13 | L-Methionine | | 44 | Phosphorylethanolamine | 75 | N, N-Dimethylglycine | |
| 14 | L-Isoleucine | | 45 | Anserine | 76 | 4-Acetamidobutyric Acid | |
| 15 | L-Proline | | 46 | Trans-4-Hydroxy-L-Proline | 77 | L-Carnosine | |
| 16 | L-Serine | | 47 | D-Homocysteine | 78 | 6-Aminocaproic Acid | |
| 17 | L-Tryptophan | | 48 | α-Aminoadipic acid | 79 | 3-Chloro-L-Tyrosine | |
| 18 | L-Phenylalanine | | 49 | L-Ornithine | 80 | S-(5-Adenosyl)-L-Homocysteine | |
| 19 | L-Tyrosine | | 50 | L-tyrosine methyl ester | 81 | Kynurenic Acid | |
| 20 | L-Cysteine | | 51 | 2-Aminobutyric acid | 82 | N'-Formylkynurenine | |
| 21 | L-Glutamic acid | | 52 | (5-L-Glutamyl)-L-Alanine | 83 | Urea | |
| 22 | L-Aspartate | | 53 | 3-Iodo-L-Tyrosine | 84 | argininosuccinic acid | |
| 23 | L-Asparagine Anhydrous | | 54 | P-Aminohippuric Acid | 85 | 5-Hydroxylysine | |
| 24 | L-Glutamine | | 55 | Glycyl-L-Proline | 86 | O-Phospho-L-Serine | |
| 25 | L-Lysine | | 56 | Trimethylamine N-Oxide | 87 | N-Acetylaspartate | |
| 26 | L-Histidine | | 57 | 1,3,7-Trimethyluric Acid | 88 | L-Homocystine | |
| 27 | L-Arginine | | 58 | 3-Hydroxyhippuric Acid | 89 | 3-Aminoisobutanoic Acid | |
| 28 | L-Threonine | | 59 | N8-Acetylspermidine | 90 | Glutathione Oxidized | |
| 29 | L-Citrulline | | 60 | (S)-β-Aminoisobutyric Acid | 91 | L-α-Aspartyl-L-phenylalanine | |
| 30 | 5-Hydroxy-Tryptamine | | 61 | S-Sulfo-L-Cysteine | 92 | N-Glycyl-L-Leucine | |
| 31 | L-Homocitrulline | | 62 | Methionine Sulfoxide |  |  | |

**Table S5.** Differential Changed Amino Acids in Healthy and Inflammatory Skin Samples.

| Name | Healthy Samples  (D28_H vs D0_H) | | | Inflammatory Samples  (D28_U vs D0_U) | | | |
| --- | --- | --- | --- | --- | --- | --- | --- |
|  | **FC** | **Log2FC** | **Type** | **FC** | | **Log2FC** | **Type** |
| N-Acetylaspartate | 0.39 | -1.35 | **↓** | |  |  |  |
| (5-L-Glutamyl)-L-Alanine | 0.29 | -1.80 | **↓** | |  |  |  |
| L-Valine | 0.43 | -1.21 | **↓** | |  |  |  |
| Beta-Alanine | 0.49 | -1.03 | **↓** | |  |  |  |
| L-Asparagine Anhydrous | 0.43 | -1.22 | **↓** | |  |  |  |
| L-Alanine | 0.37 | -1.44 | **↓** | |  |  |  |
| L-Glutamine | 0.38 | -1.39 | **↓** | |  |  |  |
| L-Tryptophan | 0.46 | -1.13 | **↓** | | 0.34 | -1.54 | **↓** |
| 5-Hydroxy-Tryptamine | 2.81 | 1.49 | **↑** | | 0.29 | -1.81 | **↓** |
| 5-Aminovaleric Acid | 0.29 | -1.75 | **↓** | | 0.48 | -1.05 | **↓** |
| argininosuccinic acid |  |  |  | | 2.61 | 1.38 | **↑** |
| 6-Aminocaproic Acid |  |  |  | | 3.06 | 1.62 | **↑** |
| 2-Aminobutyric acid |  |  |  | | 0 | -lnf | **↓** |
| Anserine |  |  |  | | 0.33 | -1.60 | **↓** |

*****FC means Fold changes


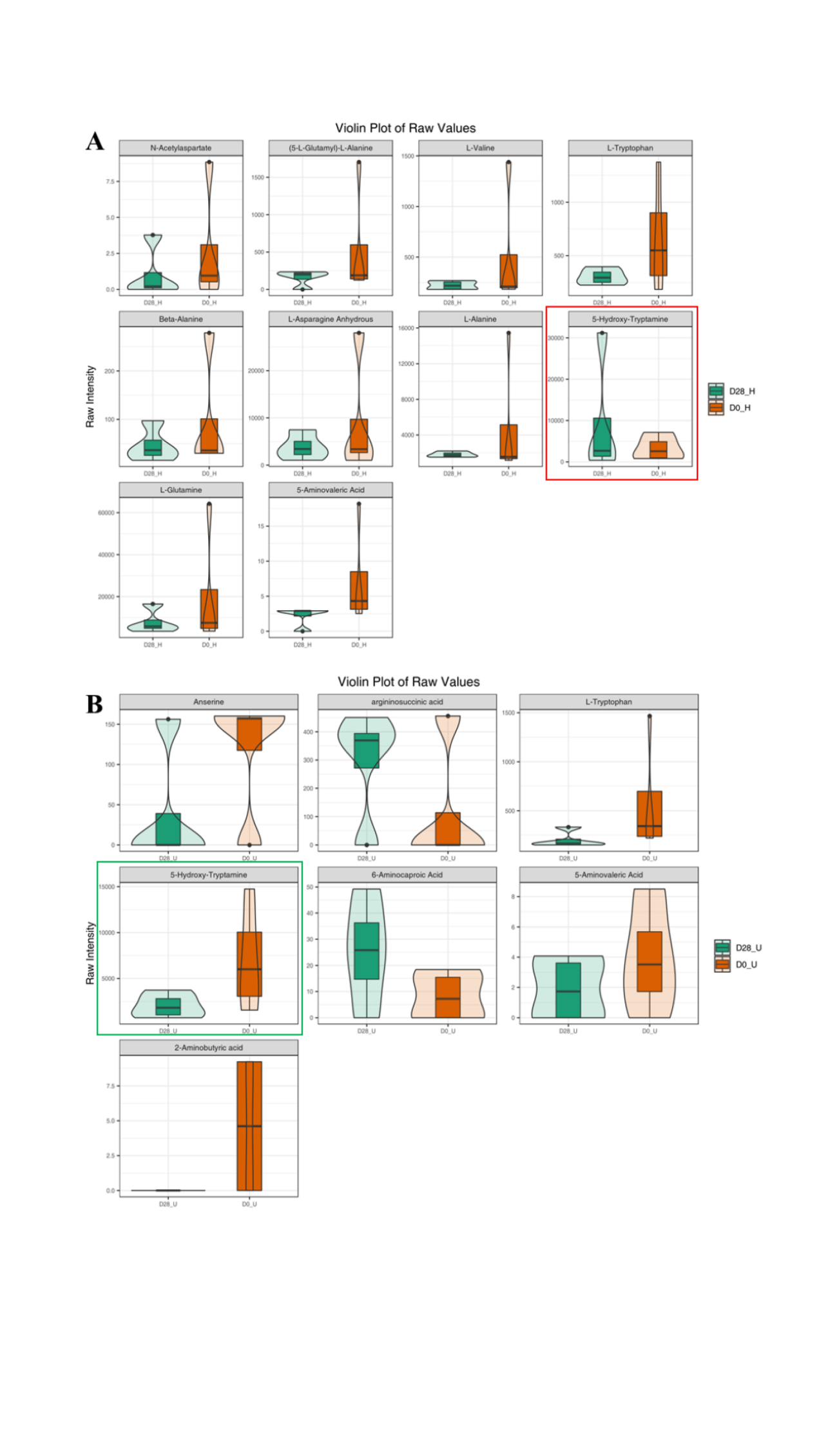


**Figure S5.** Volin plots of raw values of differential amino acids in healthy (A) and inflamed (B) skin before and after donkey oil treatment.

**Table S6.** All Detected Free Fatty Acids in This Study.

| **No.** | **Name** | **No.** | **Name** |
| --- | --- | --- | --- |
| 1 | hexanoic acid | 25 | trans-vaccenic acid |
| 2 | octanoic acid | 26 | stearic acid |
| 3 | nonanoic acid | 27 | cis-10-carboenoic acid |
| 4 | decanoic acid | 28 | nonadecylic acid |
| 5 | cis-10-undecanoic acid | 29 | hexadecanedioic acid |
| 6 | hendecanoic acid | 30 | arachidonic acid |
| 7 | lauric acid | 31 | cis-5,8,11,14,17-eicosapentaenoic acid |
| 8 | tridecanoic acid | 32 | cis-8,11,14-eicosatrienoic acid |
| 9 | myristoleic acid | 33 | cis-11,14-eicosadienoic acid |
| 10 | myristic acid | 34 | cis-11-eicosenoic acid |
| 11 | cis-10-pentadecenoic acid | 35 | cis-11,14,17-eicosatrienoic acid |
| 12 | pentadecanoic acid | 36 | trans-11-eicosenoic acid |
| 13 | cis-9-palmitoleic acid | 37 | arachidic acid |
| 14 | trans-9-palmitelaidic acid | 38 | heneicosanoic acid |
| 15 | palmitic acid | 39 | cis-4,7,10,13,16,19-docosahexaenoic acid |
| 16 | cis-10-heptadecanoic acid | 40 | cis-7,10,13,16-docosatetraenoic acid |
| 17 | trans-10-heptadecenoic acid | 41 | cis-7,10,13,16,19-docosapentaenoic acid |
| 18 | heptadecanoic acid | 42 | cis-13,16-docosadienoic acid |
| 19 | γ-linolenic acid | 43 | erucic acid |
| 20 | linoleic acid | 44 | trans-13-docosenoic acid |
| 21 | α-linolenic acid | 45 | behenic acid |
| 23 | cis-9-octadecenoic acid | 46 | tricosanoic acid |
| 22 | linolelaidic acid | 47 | nervonic acid |
| 24 | trans-9-octadecenoic acid | 48 | lignoceric acid |

**Table S7.** Differential Changed Free Fatty Acids in Healthy and Inflammatory Skin Samples.

| Name | Healthy Samples  (D28_H vs D0_H) | | | Inflammatory Samples  (D28_U vs D0_U) | | | |
| --- | --- | --- | --- | --- | --- | --- | --- |
|  | **FC** | **Log_2_FC** | **Type** | **FC** | | **Log_2_FC** | **Type** |
| decanoic acid | 0.23 | -2.10 | **↓** | | 0.12 | -3.11 | **↓** |
| octanoic acid | 0.23 | -2.14 | **↓** | | 0.05 | -4.38 | **↓** |
| cis-11,14,17-eicosatrienoic acid | Inf | Inf | **↑** | | Inf | Inf | **↑** |
| tridecanoic acid | 2.02 | 1.02 | **↑** | | 0.45 | -1.16 | **↓** |
| myristic acid | 2.27 | 1.19 | **↑** | | 0.43 | -1.20 | **↓** |
| pentadecanoic acid | 2.24 | 1.17 | **↑** | | 0.39 | -1.33 | **↓** |
| hexanoic acid | 2.05 | 1.03 | **↑** | |  |  |  |
| cis-9-octadecenoic acid | 3.33 | 1.74 | **↑** | |  |  |  |
| trans-9-octadecenoic acid | 2.28 | 1.19 | **↑** | |  |  |  |
| linoleic acid | 2.37 | 1.24 | **↑** | |  |  |  |
| lauric acid |  |  |  | | 0.39 | -1.36 | **↓** |

*****FC means Fold changes


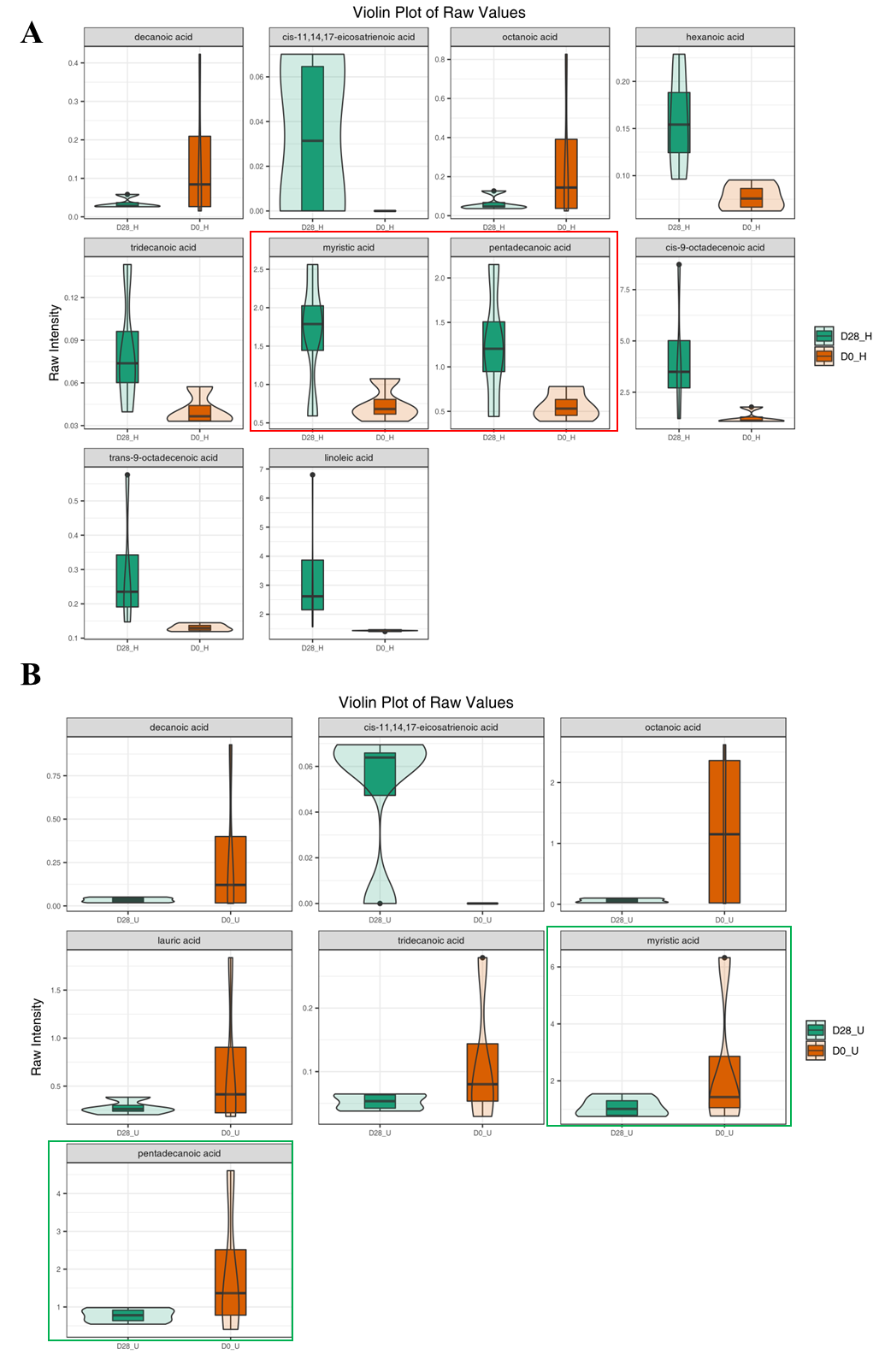


**Figure S6.** Volin plots of raw values of differential amino acids in healthy (A) and inflamed (B) skin before and after donkey oil treatment.
